# Unilateral Testicular Tuberculosis: An Extra-Pulmonary Manifestation

**DOI:** 10.7759/cureus.18896

**Published:** 2021-10-19

**Authors:** Khalid A Al-Hashimi, Umar N Said

**Affiliations:** 1 Vascular Surgery, Royal Shrewsbury Hospital, Shrewsbury, GBR; 2 General Medicine, Bradford Royal Infirmary, Bradford, GBR

**Keywords:** genitourinary tuberculosis, anti-tuberculosis therapy, scrotal infection, orchidectomy, tuberculosis, extra-pulmonary tuberculosis

## Abstract

The pathogenicity of *Mycobacterium tuberculosis (M. tuberculosis)* causes it to most commonly manifest within the respiratory system (pulmonary tuberculosis); however, 15% of cases undergo extra-pulmonary spread to various organs. Genitourinary tuberculosis (GUTB) is a rare form of tuberculosis infection which has a propensity to affect the genitourinary tract, primarily affecting the kidneys, epididymis, seminal vesicles and prostate; however, 0.5% of cases result in infection of the testicles. This may present unilaterally or bilaterally with varying atypical presentations, thus misleading physicians in diagnosis. We present a case in a 48-year-old patient admitted to the surgical assessment unit in our hospital presenting with a unilateral painful testicular lesion and scrotal changes. He was admitted nine weeks prior for unexplainable constitutional symptoms however presented again whilst awaiting follow up in an outpatient clinic. Ultrasound guidance and fine-needle aspiration & culture (FNAC) of the lesion resulted in a positive diagnosis for *M. tuberculosis*. He underwent anti-tuberculous chemotherapy treatment for six months as per clinical guidance with adequate clinical response.

## Introduction

Tuberculosis (TB) is a life-threatening disease with great prevalence in lower-income countries whereby socio-economic conditions & poor sanitation predominates. The incidence has dramatically risen over recent years due to concurrence in HIV affected populations as well as malnutrition, continuing population migration and malignancy [1]. *Mycobacterium tuberculosis* is the infective agent which leads to TB and is a part of the *Mycobacterium tuberculosis* complex; approximately 30% of the population are infected with a latent *Mycobacterium tuberculosis* infection (LTBI), with the largest rates of infection situated primarily in India accounting for 26% of global prevalence [2,3]. Since transmission of the bacterium is through inhalation of respiratory droplets [4], pulmonary tuberculosis is the most common disease sub-type of TB. *Mycobacterium tuberculosis* has a proclivity to affect the lungs first, whereas extra-pulmonary TB comprises up to 15% of cases and occurs secondary to haematogenic or lymphatic dissemination of the bacterium; it can be re-activated at a later stage resulting in symptomatic presentation distal to the lungs [5].

Genitourinary TB is a rare sub-type of extra-pulmonary tuberculosis, of which only 3% is made up by testicular TB cases [6]. The pathophysiology by which this arises has been debated, with the two key potential explanations being either haematogenous spread or local infectious spread from the seminal vesicles or the prostate via retrograde ejaculation [7]. The disease process tends to cause epididymitis in its initial stages; however, testicular involvement soon ensues. It has a propensity to affect sexually active males between the ages of 20-50, who will present typically with a painless or painful scrotal mass or swelling. A potential differential diagnosis of testicular malignancy is often contemplated as it tends to be more common than TB; however, it is often ruled out by ultrasound and fine-needle aspiration of the lesion [8]. Here we report a rare case of a gentleman presenting to the hospital with a case of testicular swelling consequently diagnosed as testicular tuberculosis.

## Case presentation

A 48-year-old male of Asian origin presented to the surgical assessment unit, following a referral by their general practitioner (GP), in light of a six week history of testicular pain and associated scrotal tenderness with no acute urinary symptoms. He described noticing the swelling as being initially painless in nature however progressively becoming increasingly painful. The pain was described as now constant with intermittent heightened episodes of burning sensations and no associated radiation. Despite GP’s provision of empirical doxycycline treatment, symptoms persisted. Upon further questioning, no additional past medical history, family history or recent travel history was discernible. Vital signs were all within regular parameters: heart rate 76, respiratory rate 18, temperature of 37.1^o^C, blood pressure of 121/78 and saturating at 98% on air. On admission, examination revealed a unilateral orchitis like picture with a 4 cm x 5 cm tender, smooth tender testicle with associated erythema of the overlying scrotum; an evident lesion could be palpated along the surface of the testicles however examination was poorly tolerated by the patient. The lesion was described as smooth, fluctuant and adherent to the surface of the testicle. Left sided inguinal lymphadenopathy was additionally noted on palpation of the abdomen. Initial workup showed grossly normal bloods (Table 1) with a white cell count of 10.8 (4.5-11 x 10^9^/L), haemoglobin of 15.3 (13.5-17.5g/dL), and platelets of 367 (150-400 x 10^9^/L). Testicular tumour markers indicated no abnormalities with an alpha-fetoprotein of <3 (0-8 kU/L), beta human chorionic gonadotropin (beta-hCG) of 1 (0-2U/L), and lactate dehydrogenase (LDH) of 194 (130-250U/L). Viral serology for hepatitis B, hepatitis C and HIV were all negative. Gonorrhea and chlamydia screening were also negative. 

**Table 1:** Blood results of the patient on admission and following further testing for testicular tumour markers Beta-hCG - beta human chorionic gonadotropin, LDH - lactate dehydrogenase

Full blood count		
Blood test	Blood result	Normal standardised range
Total white cell count	10.8	(4.5-11 x 10^9^/L)
Haemoglobin level	15.3	(13.5-17.5g/dL)
Platelet count	367	(150-400 x 10^9^/L)
Haematocrit	0.44	(0.4-0.51)
Mean cell volume	93.2	(80.0-99.0)
Mean cell haemoglobin	32.4	(27.5-32.5 pg)
Mean cell haemoglobin concentration	348	(310-350 g/L)
Red blood cell count	4.72	(4.25-6.00x 10^12^/L)
Neutrophil count	7.75	(1.7-8.0 x 10^9^/L)
Lymphocyte count	1.19	(1.0-4.0x 10^9^/L)
Monocyte count	0.8	(0.20-0.80 x 10^9^/L)
Eosinophil count	0.04	(0.04-0.40 x 10^9^/L)
Basophil count	0.02	(0.02-0.10 x 10^9^/L)
Testicular tumour marker profile		
Blood test	Blood result	Normal standardised range
Alpha-fetoprotein	<3	(0-8 kU/L)
Beta-hCG	1	(0-2 U/L)
LDH	194	(130-250 U/L)

**Figure 1:**
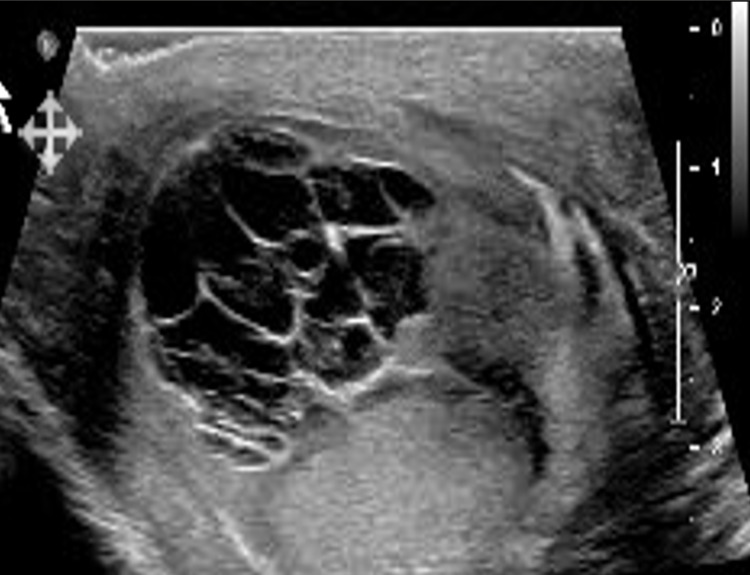
Ultrasound scan of left testicle revealing 2 cm extra-testicular lesion with multiple fluid-filled loculations

Intravenous levofloxacin was commenced for a two-week period on the basis of suspected orchitis in compliance with hospital guidance. Failure to show any resolution of symptoms and lack of response to empirical treatment warranted imaging of the testes. Ultrasound scanning (Figure 1) showed a multi-loculated heterogenous extra-testicular collection along the surface of the ipsilateral teste measuring approximately 2.1 cm in diameter. A diagnosis of complex orchitis was still considered however it was suggested the lesion could be of potential malignant nature. Fine-needle aspiration yielded 11 millilitres of fluid from the affected testicle. This fluid subsequently underwent culturing and polymerase chain reaction testing, confirming the presence of *Mycobacterium tuberculosis*. Considerations were taken as to whether a surgical approach would be favourable as opposed to the use of anti-tuberculous chemotherapy regimes. A decision was made to initiate standard quadruple therapy in concurrence with guidance. A six-month-long regime was commenced consisting of rifampicin 600 mg daily and isoniazid 300 mg daily alongside pyrazinamide 2 g daily and ethambutol 1300 mg daily; the latter two were discontinued after a two month period as per recommendations. Avoidance of orchidectomy was achieved following satisfactory clinical response to medical treatment; gradual resolution of painful swelling with normalisation of surrounding scrotal skin was noted.

**Figure 2:**
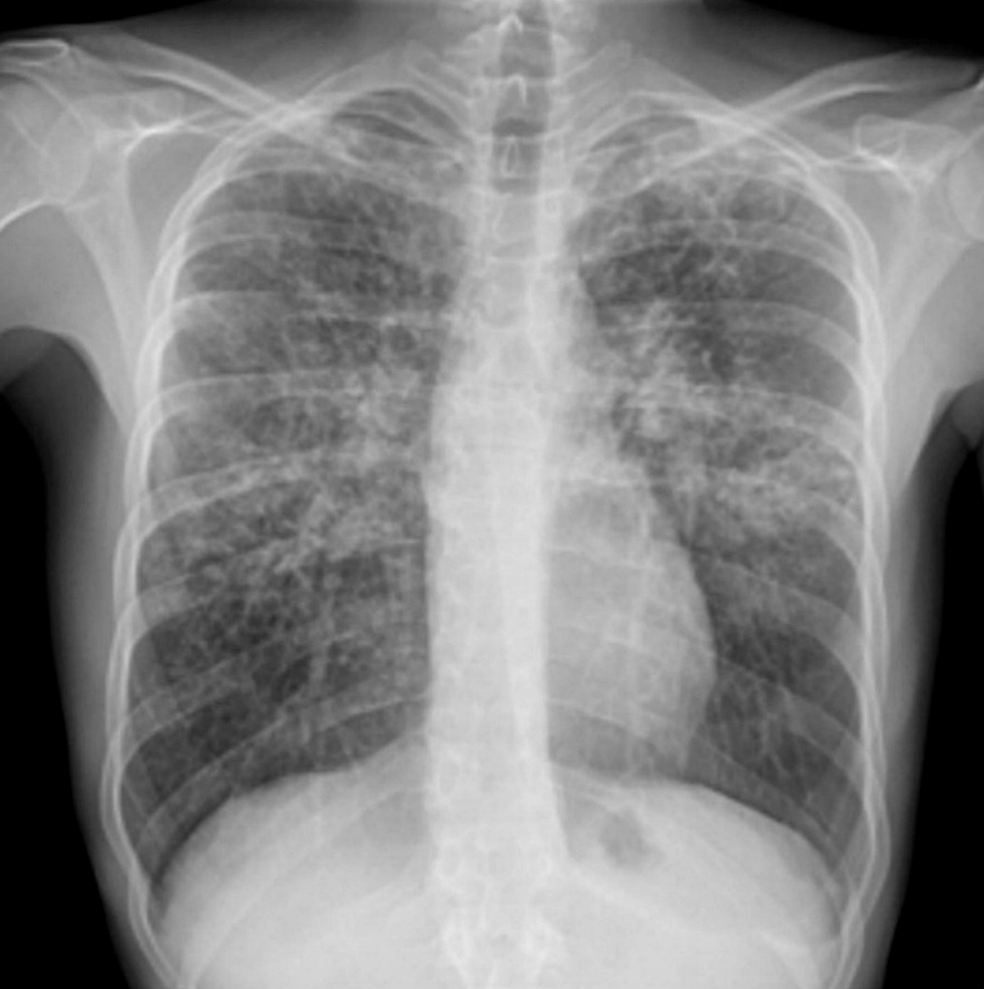
Chest X-Ray nine weeks prior to admission revealing bilateral hilar lymphadenopathy

**Figure 3:**
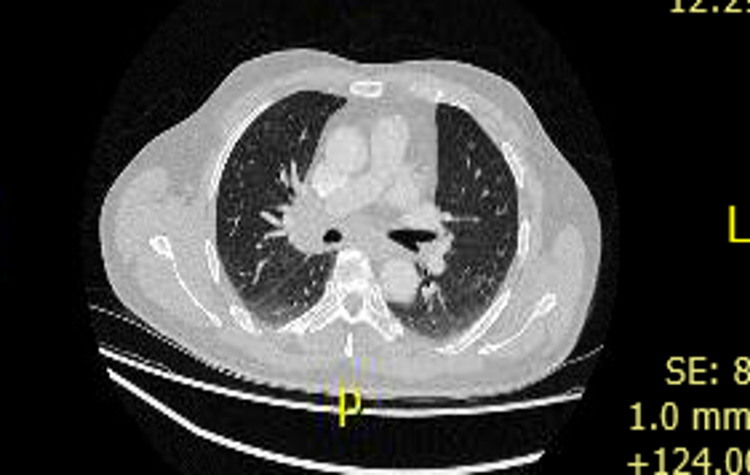
Computerised tomography on second admission to assess for pulmonary cavitations

Upon inspection of previous hospital records, it was noted that the patient had been admitted nine weeks prior to onset of his testicular discomfort for non-specific general malaise, lethargy, and weight loss. The patient volunteered that he had noticed this prior to the onset of testicular symptoms. A chest X-ray was performed exhibiting bilateral hilar lymphadenopathy thus potential diagnoses of tuberculosis, sarcoidosis and lymphoma were considered (Figure 2). A negative serum angiotensin-converting enzyme (ACE) of <40 µg/L resulted in the need for biopsy, revealing necrotic collections on the histology. Acid fast bacillus (AFB) cultures were performed however no growth of *Mycobacterium tuberculosis* was isolated; he was asked to return for in house respiratory follow up as an outpatient clinic appointment. In light of confirmation of the diagnosis following the second presentation, a CT of the thorax was performed to assess for further cavitation within the lungs (Figure 3).

## Discussion

Genitourinary TB was initially discovered and coined by Willbolz et al. [9]. Initially believed to be the most common subtype of extra-pulmonary TB, recent research has indicated that it constitutes less than 0.5% of extra-pulmonary TB in developed countries [10]. It has a wide array of presentations, of which the most commonly affected site is the kidneys, followed by epididymis, seminal vesicles, prostate, testis, and lastly, vas deferens [11]. The presentation of the disease is often unclear, and more common differential diagnoses are often considered prior to an incidental diagnosis of tuberculosis; testicular teratomas and seminomas being more common presentations of testicular swelling poses some difficulty distinguishing testicular TB from malignancies [12].

 Large variations in presentation have been noted in literature. Cases may only present with a non-specific testicular nodule whilst others suffer with additional constitutional symptoms similar to our case, such as malaise, fever, and weight loss in the complete absence of urinary symptoms [13]. Physical examination findings are also of benefit as scrotal thickening and epididymal enlargement are commonly associated with infection alongside either a painful or painless testicular mass [14]. Most cases of testicular tuberculosis are responsive to anti-tuberculous chemotherapy; however, atypical presentations still pose an issue, commonly leading to unnecessitated orchidectomies whereby medical therapies alone were likely to be sufficient [15].

The utilisation of ultrasound and fine-needle aspiration cytology (FNAC) as further diagnostic testing may enable clinicians to come to a more accurate diagnosis earlier in the patient’s care, thus allowing avoidance of early surgical approaches [15]. This was displayed in this case as we intended to adopt a more investigative approach, thus saving a patient from risks of iatrogenic infertility as well as the need for future post-resection counselling.

## Conclusions

Though testicular tuberculosis may be deemed a rare presentation and an unlikely diagnosis, it is one that should be considered within patients aged 20-50 presenting with a painful testicular mass. Testicular neoplasms are the more common presentation within similar age groups; however, this case emphasises the benefits of employing the use of more thorough diagnostic measures. These may result in a reduction in the risk of provision of unnecessary intervention to patients; clinicians should also be aware as well as suspect this diagnosis in antibiotic-resistant scrotal infections in the absence of urinary symptoms.
